# Findings of the Resident Workload Using Mobile Application in Japan

**DOI:** 10.31662/jmaj.2024-0094

**Published:** 2024-11-11

**Authors:** Saki Muroya, Sachiko Ohde, Takako Morita, Seisyou Kou, Yosuke Homma, Joshua L. Jacobs, Gautam A. Deshpande

**Affiliations:** 1 Graduate School of Public Health, St. Luke’s International University, Tokyo, Japan; 2 Research Institute for Medical Education, St. Marianna University School of Medicine, Kawasaki, Japan; 3 Department of Emergency Medicine, Chiba Kaihin Municipal Hospital, Chiba, Japan; 4 Department of Medical Education and Clinical Sciences, Elson S. Floyd College of Medicine at Washington State University, Spokane, U.S.A; 5 Department of International Healthcare, Juntendo University Hospital, Tokyo, Japan; 6 Department of Internal Medicine, John A. Burns School of Medicine, University of Hawaii, Honolulu, U.S.A

**Keywords:** Residency, Workload, Japan, Mobile app, Work-life balance

## Abstract

**Introduction:**

Excessive workload among medical residents remains a social issue, particularly in Japan. The government requires management of overtime work in health institutions. Among young healthcare workers, the demand for sustainable work-life balance is increasing. This study evaluated the current workload and work allocation of postgraduate residents using a mobile application.

**Methods:**

A cross-sectional study including postgraduate trainees from three major teaching hospitals was conducted in 2021 using a mobile application. The residents recorded their work (direct patient care, indirect patient care, education, research, administration, personal time, and others) using the application. The data were descriptively analyzed.

**Results:**

A total of 69 residents participated in the survey. Their mean working hours was 11 h and 45 min, and their mean sleep time was 6 h and 18 min. The proportions of work allocation time by category were 35.5% for direct patient care; 35.5%, indirect patient care; 10.1%, personal time; 9.4%, education; 8.6%, administration; and 1%, research.

**Conclusions:**

The development of a mobile application enabled us to measure the residents’ workload and work allocation. The time spent on direct and indirect patient care increased over a decade, whereas the time spent on educational activities and research remained limited.

## Introduction

Excessive workload among young physicians, such as residents, is a social issue that has been observed in numerous countries ^(1), (2)^, including Japan ^(3)^. Previous studies have demonstrated that sleep deprivation and long work hours are prevalent among residents ^(4), (5), (6)^. Burnout, depression, and death from overwork among residents have also been reported in the context of the revision of postgraduate medical training ^(5), (7), (8), (9), (10), (11)^.

In Japan, a multispecialty rotation curriculum has been mandated by the Japanese government since 2004 for all residents in their first 2 years after graduating from medical school. This curriculum was introduced to provide the opportunity for physicians to comprehensively experience various subspecialties before deciding a professional career. While numerous studies have reported that residents’ basic clinical competencies improved after the implementation ^(12), (13), (14)^, others have reported the potential impact on the amount of residents’ work and their quality of life ^(15)^. Recent studies have demonstrated that a quarter to half of residents experience burnout during their residency program ^(16), (17), (18), (19)^, and some even suffer from depression ^(17), (19)^. Postgraduate training provides an appropriate salary to residents. Hence, residents in Japan are not allowed to work part-time outside of training. Nevertheless, long working hours remain an issue and are among the contributing factors to burnout in residents ^(16), (17)^.

To address the issues arising from long working hours across all industries, the Japanese government introduced a new policy of work style reforms in 2017, and the regulation of overtime work by physicians was started in April 2024 ^(20)^. In response to this new regulation, all health institutions were advised to monitor and control overtime work by all physicians in Japan, including residents, during the grace period. Work-life balance is considered to be important by the government and by young healthcare workers who tend to choose jobs that allow them to achieve balance. Nakayasu et al. found that residents consider work-life balance to be more important than their medical interest, and they may choose their specialty because of social reasons regardless of their professional interest ^(20)^. The management of work-life balance rather than only overtime work is critical for health institutions ^(21)^.

In 2012, Deshpande et al. explored work-life balance before several of these new regulatory changes were implemented and found detailed work allocation and sleep deprivation among residents ^(22)^. To more effectively implement work policy and achieve work-life balance for residents, a measurement of this issue needs to be revisited. This study aimed to measure the current workload and work allocation of postgraduate year (PGY) 1 and 2 residents using a mobile application ^(22)^. Based on these findings, we can gain insight into how to reform residents’ work styles ^(21)^.

## Materials and Methods

### Study design, setting, and sample

We developed a mobile application that enables residents to easily record working hours and the type of work in which they engage (work content) via smartphone. A cross-sectional study using this mobile application was conducted at three major teaching hospitals in Japan, namely, St. Luke’s International Hospital in Tokyo, St. Marianna University School of Medicine Hospital in Kanagawa, and Tokyo Bay Urayasu Ichikawa Medical Center in Chiba, which were anonymized in the results, where work hours were strictly enforced. All postgraduate trainees (PGY 1 and 2) working at these hospitals were invited to participate in the study. Before the study initiation, an orientation was conducted at each site to explain the purpose of the study and how to record work allocation using smartphones. All the participants provided written informed consent and were provided a smartphone for the study. Furthermore, they were compensated with a JPY 5,000 gift card. IRB approval was obtained for each institution (20-R183).

### Development of software

We developed mobile application software for this study that enabled residents to record their work content on their smartphones. The residents were asked to carry the smartphones during their working time. The smartphone alarm sounded every hour, and the residents were asked to record what they had done in the past hour using an interactive chart. Work content was categorized into (1) direct patient care, (2) indirect patient care, (3) education, (4) research, (5) administration, (6) personal time, and (7) others. These categories were based on our previous study ^(22)^, where we confirmed that the majority of the work content by residents could be categorized into those items. Furthermore, we provided three blank categories so that the residents could freely create a new category if their work content could not be categorized into an existing one. The detailed categorization of work content has been defined elsewhere ^(22)^. Briefly, direct patient care included explaining to and counseling patients and/or families about their condition and treatment, conducting physical examinations and tests, visiting patients’ rooms, and performing surgeries, including endoscopy and intubation. Indirect patient care included checking test results, consulting with specialists and/or senior physicians, charting patient information, and organizing the treatment schedule. Education included preparing for and/or attending study groups, conferences, and self-study. Research included data collection for presentations, attending academic conferences, and preparing case reports. Administration included committee activities and tasks related to hospital management. Finally, personal time included time for meals and sleeping, time with family and friends, and Internet use for personal purposes. We provided a small card with the list of activities in each category so that the residents could refer to it during the survey.

### Data collection

The survey was conducted from February to March 2021 and included residents from three institutions in Japan who worked both the day and night shifts. The same number of residents was randomly assigned each day during the calendar week at each institution. The residents were asked to turn on the phone on their assigned day and start recording their workload at their clock in. They carried their phones throughout their shift. The phones were set to provide a reminder (an alarm or vibration) every hour, at which time the residents were asked to record their work allocation for the previous hour. If the residents were unable to record at the time they were reminded, they were allowed to record the information later. The data collection was finished when the residents clocked out. During the survey period, we also organized a technical support desk for residents who encountered technical problems. We also collected demographic data and self-reported sleep data for the subanalysis.

### Statistical analysis

The data were anonymized, and descriptive statistics were used to characterize the residents by PGY, gender, age, reported work hours, and sleep data. The chi-squared test was employed to compare the proportions for the categorical variables, and one-way analysis of variance was used to compare means among the institutions. Subsequently, the data were descriptively analyzed for the time spent in a 24-h period for each of the seven categories: (1) direct patient care, (2) indirect patient care, (3) education, (4) research, (5) administration, (6) personal time, or (7) other. All analyses were conducted using IBM SPSS Statistics (version 27) and Stata Corp. 2019 (Stata Statistical Software: Release 16. College Station, TX: StataCorp LLC).

## Results

The survey was conducted from February to March 2021. No residents at any institution reported technical trouble or any need to seek support during the survey period. A total of 69 residents participated in the survey (22, 34, and 13 residents from hospitals A, B, and C, respectively). Table 1 presents the residents’ characteristics and response rates for each category. As can be seen from the table, 36 and 33 part were PGY 1 and PGY 2, respectively. Their mean age was 27.2 years old, and 65% of them were men. Their mean working hours was 11 h and 45 min, and their mean sleep time was 6 h and 18 min. No statistically significant differences were observed in the residents’ demographic characteristics and mean working hours among the participating hospitals. However, the mean sleep time was approximately 60 min shorter at Hospital A than in other hospitals.

**Table 1.** Characteristics of Residents among the Hospitals.

	Total	Hospital A	Hospital B	Hospital C	*P*-value
		(N = 22)	(N = 34)	(N = 13)	
PGY-1, N (%)	36 (52)	11 (50)	17 (50)	8 (61.5)	0.755
Gender (Male), N (%)	45 (65)	15 (68.2)	23 (68)	7 (54)	0.633
Age, Mean (SD)	27.2 (2.2)	27.1 (2.6)	27.6 (2.1)	26.2 (1.2)	0.07
Work hours, Mean (SD)	11 h 45 mins (275 mins)	12 h 35 mins (377 mins)	10 h 43 mins (212 mins)	13 h 5 mins (185 mins)	0.171
Sleep time, Mean (SD)	6 h 18 mins (57 mins)	5 h 38 mins (44 mins)	6 h 36 mins (50 mins)	6 h 38 mins (62 mins)	0.001*
# of residents who recorded each work allocation					
Direct care	69 (100%)	22 (100%)	34 (100%)	13 (100%)	-
Indirect care	69 (100%)	22 (100%)	34 (100%)	13 (100%)	-
Education	42 (60.9%)	11 (50.0%)	22 (64.7%)	9 (69.2%)	0.431
Research	7 (10.1%)	3 (13.6%)	0 (0.0%)	4 (30.8%)	0.006*
Administration	41 (59.4%)	13 (59.1%)	19 (55.9%)	9 (69.2%)	0.706
Personal time	59 (85.5%)	18 (81.8%)	29 (85.3%)	12 (92.3%)	0.695

*One-way ANOVA

**Figure 1.**
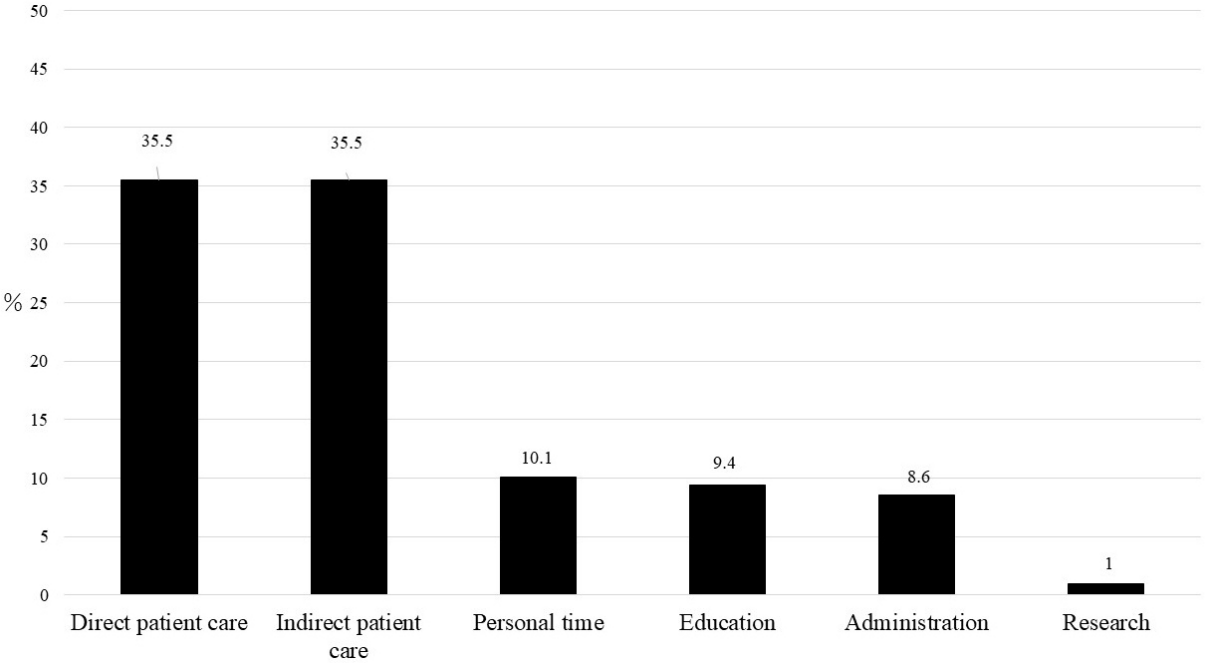
Residents’ work allocation.

Direct and indirect care were recorded by the residents. Only 60.9% of them participated in educational activities and 59.4% participated in administrative activities. These numbers varied between hospitals. Furthermore, only 10.1% recorded research-related activities. The proportion of work allocation time that was spent in each category is summarized in Figure 1. The most time was used for direct and indirect patient care (35.5% for both), followed by personal time (10.1%), education (9.4%), administration (8.6%), research (1%), and others (0%).

**Figure 2.**
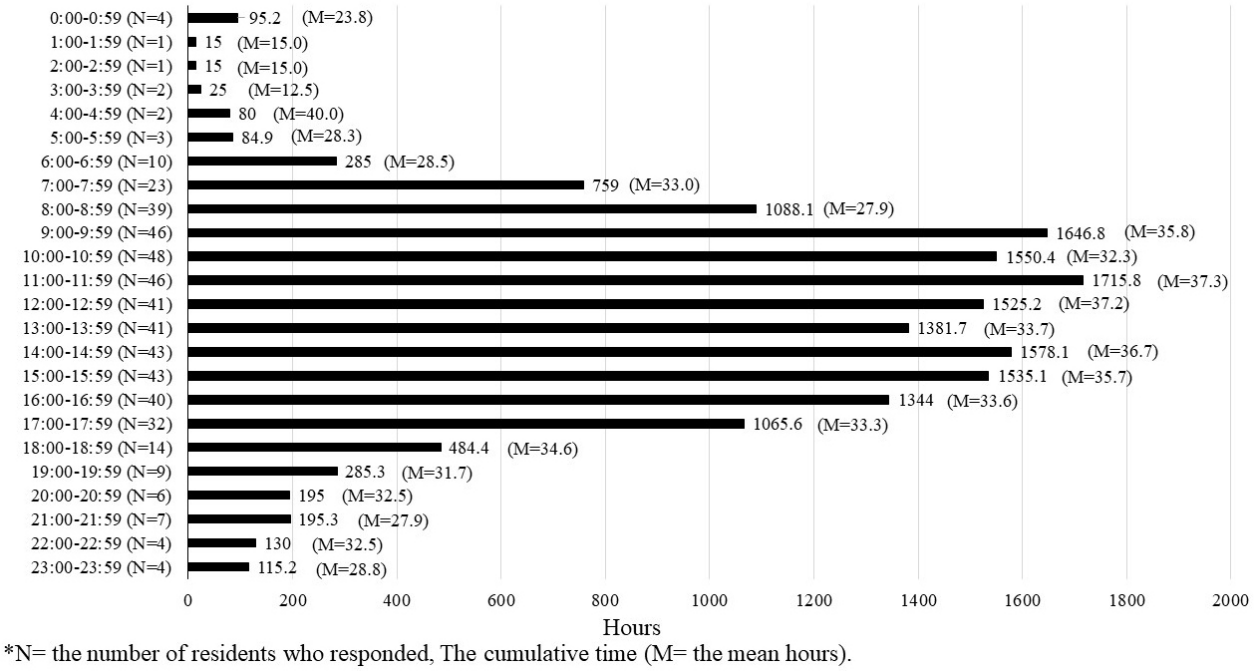
The cumulative time spent by residents on direct patient care.

**Figure 3.**
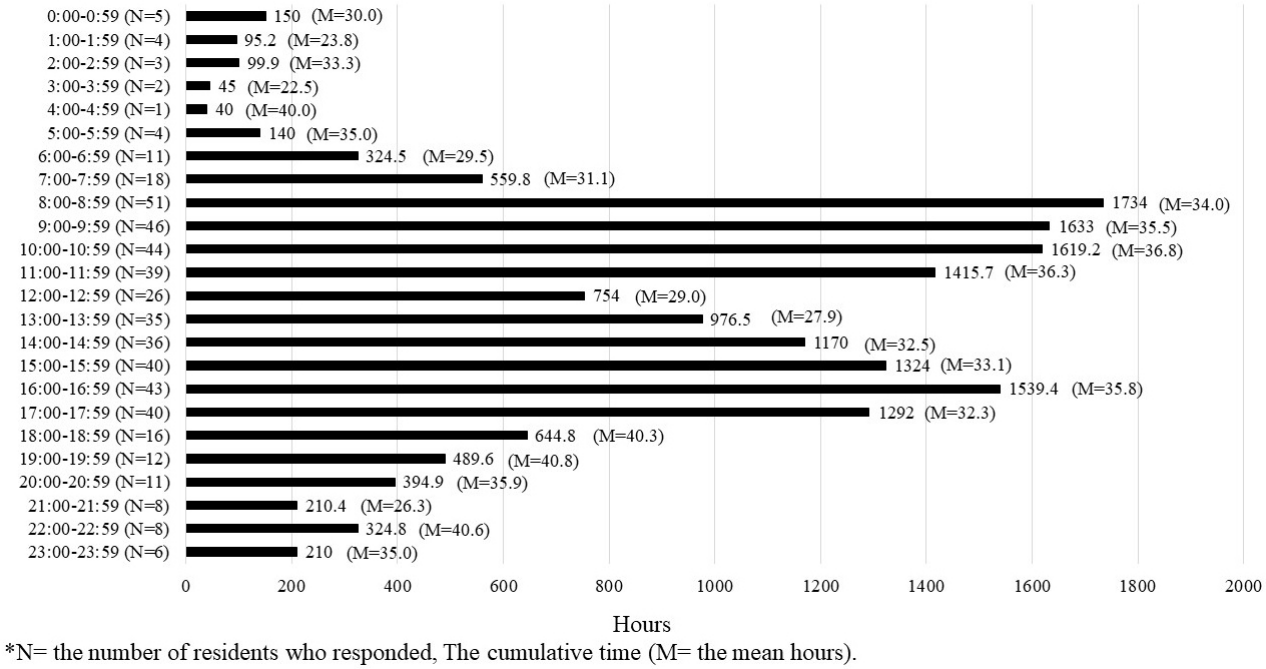
The cumulative time spent by residents on indirect patient care.

**Figure 4.**
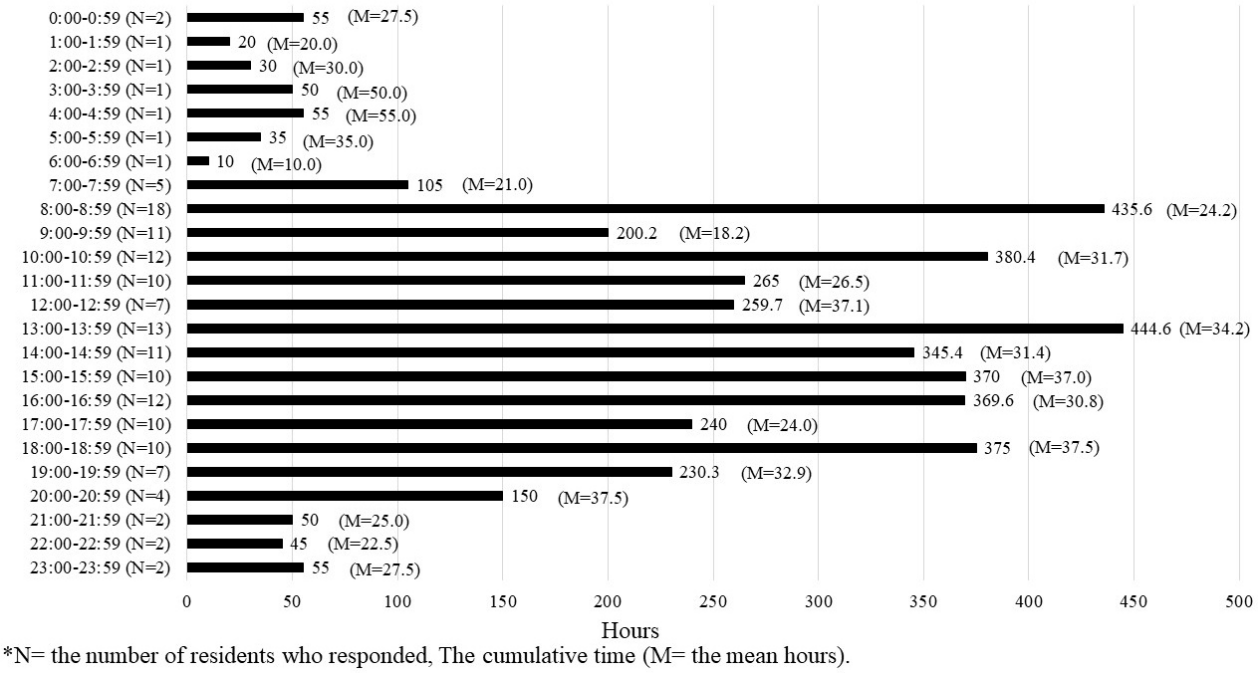
The cumulative time spent by residents on education.

Figure 2, Figure 3, and Figure 4 present the cumulative time spent by residents on shifts across all categories represented in a 24-h period. The number of residents who responded during that time frame is shown as N, whereas the mean time in that time frame is shown as M. The time spent on direct and indirect patient care was found throughout the day shift. Indirect patient care was observed even after hospital hours and sometimes during the night shift. The time spent on educational activity was found throughout the day to evening hours and at night after hospital hours.

## Discussion

The present study monitored recent residents’ workload and work allocation using a mobile application. The results indicated that residents spent approximately 71% of their work time on direct and indirect patient care, representing an approximately 10% increase from our previous study in 2012 ^(22)^. One possible reason is that Japan has been experiencing an ultra-aging society, which puts a greater burden on medical care providers caring for elderly patients who have multiple medical issues and require more attention and time for explanations for their treatments. In addition to ultra-aging, the influence of the COVID-19 outbreak that occurred during the study period likely increased the residents’ time spent on patient care.

The time spent on education (9.4%) increased by approximately 6% from 2012. This could be the result of development in the educational system during work hours. Vucicevic et al. ^(23)^ found that duty hour restrictions lead to higher attendance rates at educational conferences. Other authors have found that a reduction in work hours contributes to more reading time for residents ^(24), (25), (26)^. Regulation of work hours does not necessarily shorten the time spent on educational activities but may exert more productive effects. However, the reported time used for education was limited to 50%-60% of the residents in each institution. Considering that the purpose of the residency program is to provide young physicians with clinical education, this result can be addressed to improve the amount of residents’ educational activities during their working hours. Takahashi et al. (2009) found that scholarly activities were associated with residents’ job satisfaction ^(27)^. By monitoring their workload, developing a systematic program that allows young physicians to be exposed to scholarly activities such as research and educational time within the hours of their residency may allow them to experience clinical satisfaction and carry that on to their later professional lives.

The mean sleep time of residents in our study was 6 h and 18 min, which drastically increased from the mean of 4.8 h in 2012. Other recent studies reported mean sleep hours among residents as close to 5.8-6 h ^(15), (18)^. These findings suggest that at present, residents are able to better manage their sleep time compared with a decade ago. However, the appropriate number of sleep hours for young adults and adults is between 7 and 9 h, according to the National Sleep Foundation ^(28)^. The development of a better work-life balance for residents may help increase their sleep hours.

This study has several limitations. First is the influence of the COVID-19 outbreak. The participating teaching hospitals took on roles as critical care medical centers to treat patients with COVID-19. Some limitations to residents’ training, such as the inability to train in rural medicine or to attend surgical sites, were also observed due to the outbreak ^(29)^. However, the development of software to measure residents’ workload was an innovative trial that enabled us to conduct the study even during the pandemic. Second, as the study was subjectively measured, we provided an alarm on the smartphone every hour to minimize self-report bias. In 2012, residents changed activities approximately every 20 min ^(22)^; thus, hour intervals may not accurately reflect their exact workload. The timing of the alarm may require consideration in future research. Furthermore, the study was conducted in three teaching hospitals located in Tokyo metropolitan area (Tokyo, Kanagawa, and Chiba), where a “healthier” work-life balance is more recognized than in other jurisdictions. Including teaching hospitals in other prefectures over the country and both community and university hospitals will provide more accurate data and reflection of the residents’ current workload. The government is supportive of improving work-life balance; thus, additional studies are expected to use this mobile application to monitor residents’ workload and provide insight into the development of resident training programs.

### Conclusions

The development of a mobile application enabled us to measure residents’ workload and work allocation. Our data indicated that residents spent approximately 70% of their work time on direct and indirect patient care, which increased over the last decade. However, the time spent on educational activities and research remained limited. Developing a systematic program that provides young physicians with more exposure to scholarly activities during residency hours may allow residents to maintain their work-life balance and satisfy their clinical experience and requirements.

## Article Information

### Conflicts of Interest

None

### Sources of Funding

This work was supported by the Ministry of Health, Labor and Welfare of Japan under Grand 201903013A “Research for formulating support for seamless medical education before and after graduation, utilizing ICT.”

### Acknowledgement

This study was supported in part by grant 201903013A, “Research for formulating support for seamless medical education before and after graduation, utilizing ICT,” from the Ministry of Health, Labor, and Welfare of Japan. The funder had no role in the design of the study, the collection, management, analysis, and interpretation of the data, the preparation, review, or approval of the manuscript, or the decision to submit the manuscript for publication. We greatly appreciate Dr. Osamu Takahashi for his professional support and co-operation with our study.

### Author Contributions

Study design: SM, TM, and SO. Data collection: SM, TM, and SO. Statistical analysis: SM, TM, and SO. SM, TM, SO, and JJ interpreted the data. GD, JJ provided clinical and statistical advice. First draft: SM. All authors revised the manuscript and approved the final version prior to submission.

### Approval by Institutional Review Board (IRB)

Ethical approval was obtained from the Research Ethics Committee of St. Luke’s International Hospital, Tokyo, Japan (approval code: 20-R183). All participants provided written informed consent for the study.

### Informed Consent

All participants provided written informed consent for the study.

## References

[1] Imrie KR, Frank JR, Parshuram CS. Resident duty hours: past, present, and future. BMC Med Educ. 2014;14 Suppl 1(Suppl 1):S1. 25559868 10.1186/1472-6920-14-S1-S1 PMC4304261

[2] Pastores SM, O’Connor MF, Kleinpell RM, et al. The Accreditation Council for Graduate Medical Education resident duty hour new standards: history, changes, and impact on staffing of intensive care units. Crit Care Med. 2011;39(11):2540-9. 21705890 10.1097/CCM.0b013e318225776f

[3] Okai T, Kawahito H, Chiba Y, et al. KowareyukuIshitachi. Iwanami Shoten; 2008. Japanese.

[4] Kalmbach DA, Arnedt JT, Song PX, et al. Sleep disturbance and short sleep as risk factors for depression and perceived medical errors in first-year residents. Sleep. 2017;40(3):zsw073. 28369654 10.1093/sleep/zsw073 PMC6084763

[5] Sen S, Kranzler HR, Didwania AK, et al. Effects of the 2011 duty hour reforms on interns and their patients: a prospective longitudinal cohort study. JAMA Intern Med. 2013;173(8):657-62. 23529201 10.1001/jamainternmed.2013.351 PMC4016974

[6] Arora VM, Georgitis E, Woodruff JN, et al. Improving sleep hygiene of medical interns: can the sleep, alertness, and fatigue education in residency program help? Arch Intern Med. 2007;167(16):1738-44. 17846392 10.1001/archinte.167.16.1738

[7] Rosen IM, Gimotty PA, Shea JA, et al. Evolution of sleep quantity, sleep deprivation, mood disturbances, empathy, and burnout among interns. Acad Med. 2006;81(1):82-5. 16377826 10.1097/00001888-200601000-00020

[8] Collier VU, McCue JD, Markus A, et al. Stress in medical residency: status quo after a decade of reform? Ann Intern Med. 2002;136(5):384-90. 11874311 10.7326/0003-4819-136-5-200203050-00011

[9] Biaggi P, Peter S, Ulich E. Stressors, emotional exhaustion and aversion to patients in residents and chief residents-what can be done? Swiss Med Wkly. 2003;133(23-24):339-46. 12923685 10.4414/smw.2003.10134

[10] Hiyama T, Yoshihara M. New occupational threats to Japanese physicians: karoshi (death due to overwork) and karojisatsu (suicide due to overwork). Occup Environ Med. 2008;65(6):428-9. 10.1136/oem.2007.037473 18487428

[11] Williams D, Tricomi G, Gupta J, et al. Efficacy of burnout interventions in the medical education pipeline. Acad Psychiatry. 2015;39(1):47-54. 25034955 10.1007/s40596-014-0197-5

[12] Nomura K, Yano E, Aoki M, et al. Improvement of residents’ clinical competency after the introduction of new postgraduate medical education program in Japan. Med Teach. 2008;30(6):e161-9. 18608959 10.1080/01421590802047307

[13] Ohde S, Deshpande GA, Takahashi O, et al. Differences in residents’ self-reported confidence and case experience between two post-graduate rotation curricula: results of a nationwide survey in Japan. BMC Med Educ. 2014;14:141. 25016304 10.1186/1472-6920-14-141 PMC4105122

[14] Muroya S, Ohde S, Takahashi O, et al. Differences in clinical knowledge levels between residents in two post-graduate rotation programmes in Japan. BMC Med Educ. 2021;21(1):226-0. 33882929 10.1186/s12909-021-02651-6 PMC8059995

[15] Ogawa R, Seo E, Maeno T, et al. The relationship between long working hours and depression among first-year residents in Japan. BMC Med Educ. 2018;18(1):50. 29587738 10.1186/s12909-018-1171-9 PMC5870810

[16] Kijima S, Tomihara K, Tagawa M. Effect of stress coping ability and working hours on burnout among residents. BMC Med Educ. 2020;20(1):219. 32660575 10.1186/s12909-020-02134-0 PMC7359507

[17] Matsuo T, Takahashi O, Kitaoka K, et al. Resident burnout and work environment. Intern Med. 2021;60(9):1369-76. 33281158 10.2169/internalmedicine.5872-20 PMC8170257

[18] Nishimura Y, Miyoshi T, Obika M, et al. Factors related to burnout in resident physicians in Japan. Int J Med Educ. 2019;10:129-35. 31272084 10.5116/ijme.5caf.53ad PMC6766397

[19] Miyoshi R, Matsuo H, Takeda R, et al. Burnout in Japanese residents and its associations with temperament and character. Asian J Psychiatr. 2016;24:5-9. 27931906 10.1016/j.ajp.2016.08.009

[20] Nakayasu A, Kido M, Katoh K, et al. Survey on specialty preference and work-life balance among residents of Japanese Red Cross hospitals. JMA J. 2020;3(2):118-24. 33150243 10.31662/jmaj.2019-0013 PMC7590388

[21] Toscano F, O’Donnell E, Broderick JE, et al. How physicians spend their work time: an ecological momentary assessment. J Gen Intern Med. 2020;35(11):3166-72. 32808212 10.1007/s11606-020-06087-4 PMC7661623

[22] Deshpande GA, Soejima K, Ishida Y, et al. A global template for reforming residency without work-hours restrictions: Decrease caseloads, increase education. Findings of the Japan Resident Workload Study Group. Med Teach. 2012;34(3):232-9. 22364456 10.3109/0142159X.2012.652489

[23] Vucicevic D, Mookadam F, Webb BJ, et al. The impact of 2011 ACGME duty hour restrictions on internal medicine resident workload and education. Adv Health Sci Educ Theory Pract. 2015;20(1):193-203. 24916955 10.1007/s10459-014-9525-5

[24] Mir HR, Cannada LK, Murray JN, et al. Orthopaedic resident and program director opinions of resident duty hours: a national survey. J Bone Joint Surg Am. 2011;93(23):e1421-9. 22159864 10.2106/JBJS.K.00700

[25] Immerman I, Kubiak EN, Zuckerman JD. Resident work-hour rules: a survey of residents’ and program directors’ opinions and attitudes. Am J Orthop (Belle Mead NJ). 2007;36(12):E172-9. 18264560

[26] Zuckerman JD, Kubiak EN, Immerman I, et al. The early effects of code 405 work rules on attitudes of orthopaedic residents and attending surgeons. J Bone Joint Surg Am. 2005;87(4):903-8. 15805223 10.2106/JBJS.D.02801

[27] Takahashi O, Ohde S, Jacobs JL, et al. Residents’ experience of scholarly activities is associated with higher satisfaction with residency training. J Gen Intern Med. 2009;24(6):716-20. 19396500 10.1007/s11606-009-0970-4 PMC2686770

[28] Hirshkowitz M, Whiton K, Albert SM, et al. National Sleep Foundation’s sleep time duration recommendations: methodology and results summary. Sleep Health. 2015;1(1):40-3. 29073412 10.1016/j.sleh.2014.12.010

[29] Medical Affairs Division, Medical Affairs Bureau, Ministry of Health, Labor and Welfare. Shingata-Corona-Virus-Kansensyo no eikyou niyoru Rinsyou-Kensyu-Byouin de okonau hissyushinryoukatou no toriatsukai ni tsuite. 2020 May 13.

